# Punishment Feedback Impairs Memory and Changes Cortical Feedback-Related Potentials During Motor Learning

**DOI:** 10.3389/fnhum.2020.00294

**Published:** 2020-08-05

**Authors:** Christopher M. Hill, Mason Stringer, Dwight E. Waddell, Alberto Del Arco

**Affiliations:** ^1^Kinesiology and Physical Education, Northern Illinois University, Dekalb, IL, United States; ^2^Health, Exercise Science, and Recreation Management, University of Mississippi, Oxford, MS, United States; ^3^Biomedical Engineering, University of Mississippi, Oxford, MS, United States; ^4^Department of Neurobiology and Anatomical Sciences, School of Medicine, University of Mississippi Medical Campus, Jackson, MS, United States

**Keywords:** reinforcement feedback, motor learning, motor retention, cortical potentials, EEG

## Abstract

Reward and punishment have demonstrated dissociable effects on motor learning and memory, which suggests that these reinforcers are differently processed by the brain. To test this possibility, we use electroencephalography to record cortical neural activity after the presentation of reward and punishment feedback during a visuomotor rotation task. Participants were randomly placed into Reward, Punishment, or Control groups and performed the task under different conditions to assess the adaptation (learning) and retention (memory) of the motor task. These conditions featured an incongruent position between the cursor and the target, with the cursor trajectory, rotated 30° counterclockwise, requiring the participant to adapt their movement to hit the target. Feedback based on error magnitude was provided during the Adaptation condition in the form of a positive number (Reward) or negative number (Punishment), each representing a monetary gain or loss, respectively. No reinforcement or visual feedback was provided during the No Vision condition (retention). Performance error and event-related potentials (ERPs) time-locked to feedback presentation were calculated for each participant during both conditions. Punishment feedback reduced performance error and promoted faster learning during the Adaptation condition. In contrast, punishment feedback increased performance error during the No Vision condition compared to Control and Reward groups, which suggests a diminished motor memory. Moreover, the Punishment group showed a significant decrease in the amplitude of ERPs during the No Vision condition compared to the Adaptation condition. The amplitude of ERPs did not change in the other two groups. These results suggest that punishment feedback impairs motor retention by altering the neural processing involved in memory encoding. This study provides a neurophysiological underpinning for the dissociative effects of punishment feedback on motor learning.

## Introduction

Learning motor skills relies mostly on sensory feedback (i.e., visual, proprioception). However, reinforcement feedback (i.e., reward and punishment) can also modulate motor learning (Wrase et al., 2007; Wächter et al., 2009; Abe et al., 2011; Nikooyan and Ahmed, 2014; Song and Smiley-Oyen, 2017). In fact, by using a visuomotor rotation task, many studies have demonstrated that these reinforcers produce dissociable effects on motor learning, with punishment enhancing the learning rate (adaptation) and reward increasing the retention (memory) of the motor task (Galea et al., 2015; Song and Smiley-Oyen, 2017; Quattrocchi et al., 2018). These behavioral effects suggest that reward and punishment feedbacks are differently processed by the brain and involve distinct neural pathways (Wrase et al., 2007; Hester et al., 2010; Galea et al., 2011). Yet, the effects of these motivational reinforcers on motor learning have been primarily explored at the level of behavior and no study has compared their neural correlates. By using electroencephalography (EEG), the present study determines whether reward and punishment feedbacks produce different effects on feedback-related neural activity during a visuomotor rotation task.

Visuomotor rotation is an error-based motor task commonly used to investigate how sensory and reinforcement feedback contribute to motor learning (Shabbott and Sainburg, 2010; Shadmehr et al., 2010; Izawa and Shadmehr, 2011; Schween and Hegele, 2017). In this task, subjects are required to adapt their reaching direction, trial by trial, to compensate for the environmental perturbation (visuomotor rotation). According to the proposed model, subject’s movement errors (i.e., sensory prediction errors) after each trial are used by the brain to update an internal model that predicts the sensory consequences of motor commands (Shadmehr et al., 2010; Marko et al., 2012; Leow et al., 2016). Updating (or re-mapping) the internal model relies on the cerebellum and promotes motor adaptation during the task (Mazzoni and Krakauer, 2006; Shadmehr et al., 2010; Taylor and Ivry, 2014). Reinforcement feedback also facilitates motor learning during the visuomotor rotation task (Batcho et al., 2016; Therrien et al., 2016; Codol et al., 2018; Holland et al., 2018). However, reinforcement feedback is computed by the brain as a different source of error (i.e., reward-prediction errors) that promotes motor adaptation by maximizing reward value (Izawa and Shadmehr, 2011; Torrecillos et al., 2014). The prefrontal cortex, in particular the anterior cingulate cortex (ACC), plays an important role in processing reinforcement feedback (Schuermann et al., 2012; Walsh and Anderson, 2012; Ferdinand and Opitz, 2014; Huang and Yu, 2014; Ullsperger et al., 2014).

EEG and event-related potentials (ERPs) are used to assess how the brain processes reinforcement feedback and how it relates to learning. Specifically, reward and punishment feedback produce ERPs that peak between 200 and 500 ms after feedback presentation and reflect different aspects of performance monitoring (i.e., error/correct, gain/loss, saliency) contained in the feedback (Nieuwenhuis et al., 2004; Cohen and Ranganath, 2007; San Martín, 2012; Walsh and Anderson, 2012; Ullsperger et al., 2014). Previous studies have shown that changes in the amplitude of ERPs after reward and punishment feedback can predict learning (Gehring and Willoughby, 2002; Nieuwenhuis et al., 2004; Holroyd et al., 2004; Yeung and Sanfey, 2004; Stürmer et al., 2011). However, most of these studies focused on cognitive tasks such as stimulus-response or decision-making (i.e., gambling tasks), and therefore, do not provide evidence on whether reward and punishment change brain processing during motor learning (Hajack et al., 2006). Critically, behavioral studies suggest that reward and punishment feedback produce different effects on the adaptation (learning) and the retention (memory) of a motor task (Song and Smiley-Oyen, 2017; Galea et al., 2015), which seem to engage distinct neural pathways (Hadipour-Niktarash et al., 2007; Huang et al., 2011). Yet whether these reinforcers change brain activity during the adaptation and retention of a motor task is still unclear and is the focus of our study.

We hypothesize that reward and punishment produce different changes in feedback-related neural activity during the adaptation and retention of the visuomotor rotation task, representing the distinct neural pathways used by these types of feedback. To test this hypothesis, we evaluate the amplitude of feedback-related ERPs after reward and punishment feedback during the visuomotor rotation task. Based on previous studies (Galea et al., 2015; Song and Smiley-Oyen, 2017), the visuomotor rotation task consisted of different conditions to assess both the learning and the retention of the task. Motor learning was evaluated during the Adaptation condition, which was divided into Early Learning and Late Learning. During this condition, subjects were required to adapt their performance to a 30° rotation while being provided with concurrent visual feedback (Hinder et al., 2010; Schween et al., 2014). Motor retention was evaluated during the No Vision condition. During this condition, subjects did not receive concurrent visual feedback or reinforcement feedback, and therefore their performance did depend on the retention of the motor skills learned. Subjects were divided into three groups: Punishment, Reward, and Control. The reinforcement feedback was presented as points that corresponded to monetary gain (reward) or loss (punishment) during the Adaptation condition. Two neutral vertical lines instead of points were presented to the Control group. Our results show that punishment feedback decreased motor retention and altered feedback-related ERPs, and suggest that punishment feedback impairs the neural processing involved in encoding motor memory.

## Materials and Methods

### Participants

Forty-two healthy, right-handed, adults (age range: 19–32 years, mean age ± SD: 21.91 ± 2.1 years, males: 18, females: 24) participated in this study.

Participants were classified as right-handed using the Edinburgh Handedness Scale (handedness score ± SD: 91.75 ± 9.93; Oldfield, 1971). The Behavioral Avoidance/Inhibition scales (BAS/BIS) were used to score sensitivity to reinforcement which is divided into four subcomponents (BAS FUN, BAS DRIVE, BAS REWARD RESPONSIVENESS, BIS). Further detail on these scales can be found elsewhere (Carver and White, 1994). Each participant was randomly assigned to one of three feedback groups: Reward, Punishment, or Control. All procedures of this study were approved by the University of Mississippi Institutional Review Board and all participants provided informed consent before data collection.

### Visuomotor Rotation Task

**Figure 1:**
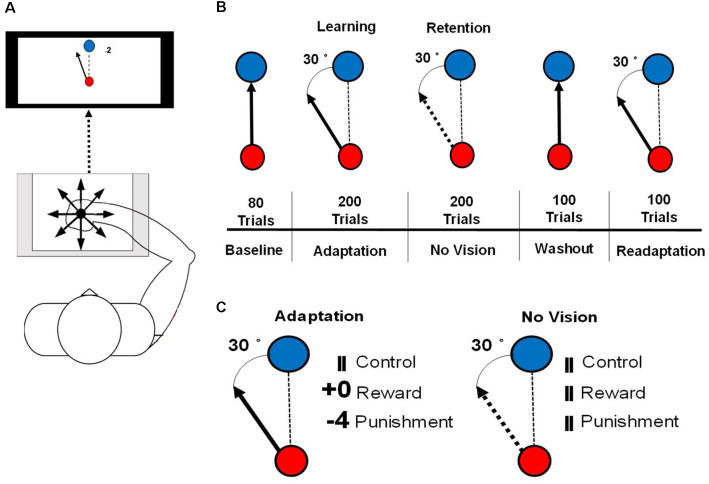
**(A)** An illustration of the set up for the motor learning task used in this study. **(B)** An illustration of the conditions of the visuomotor rotation task. The solid arrow represents the visible cursor trajectory that can be viewed by the participant. The dashed arrow represents the cursor trajectory that is not visible to the participant. The dashed line represents the direction of the cursor moved by the participant. **(C)** Reinforcement feedbacks presented during the Adaptation and No Vision conditions of the motor task. The number of points depended on the amount of error and were associated with a monetary gain (Reward) or loss (Punishment).

The visuomotor task procedures followed those outlined in Galea et al. (2015) and Song and Smiley-Oyen (2017). Participants were seated in front of a 114.3 cm television screen, at a distance of 61 cm, with a Wacom tablet and pen (sampling rate: 100 Hz) displaying two different circles (small red, large blue; Figure 1A). Trials were participant-initiated by clicking on the red starting circle after from which a line followed the movement of the cursor. The blue target circle was displayed eight centimeters from the starting circle in eight different positions, pseudo-randomly so that every set of eight consecutive trials would include one of each of the target positions.

Each participant was instructed to hold the Wacom pen in a hand posture that was most similar to writing. To limit online movement corrections, participants were instructed to move quickly and accurately, in a straight-shooting motion from the starting circle through the target with the Wacom pen. The cursor trajectory was provided 2 cm past an invisible circle boundary that passed through the center of the target circle, after which the drawn cursor trajectory was fixed and cursor movement was not available to the participant. After the presentation of the feedback, the drawn cursor trajectory, feedback, and target circle were cleared from the screen. A set of crosshairs, that followed the pen movement, was provided that allowed the participant to move accurately back to the starting circle at a self-selected pace. Each participant’s right arm was visually occluded to eliminate visual feedback during task performance. A duration criterion of 500 ms was placed on each trial, meaning that once participants initiated the trial, they had 500 ms to move their cursor past the invisible circle boundary that passes through the target circle, which is similar to previous studies (Galea et al., 2015; Song and Smiley-Oyen, 2017). If the trial was not completed within 500 ms, the trial was restarted with a message informing the participant to perform quicker. To best isolate the feedback-related neural activity from movement-related neural activity, feedback (reward, punishment, and control) was presented 1.5 s after the end of the movement, for 1 s after each trial during each testing condition of the visuomotor rotation task.

Participants performed a total of 680 trials consisting of five testing conditions: Baseline (80 trials), Adaptation (200 trials), No Vision (200 trials), Washout (100 trials), and Readaptation (100 trials; Figure 1B). After every block of 20 trials, 1 min rest period was provided and participants were instructed to keep the arm under the visual occlusion. During the Baseline and Washout conditions, target and cursor movement were congruent. Adaptation, No Vision, and Readaptation featured an incongruent position of the cursor and the target, with the cursor trajectory, rotated 30° counterclockwise to the target, requiring the participant to adapt their movement to hit the target. Points were displayed following the magnitude of the error and their assigned group (Reward and Punishment groups). Null feedback consisting of two vertical lines was presented for the Control group (Figure 1C). During the No Vision condition, reinforcement feedback and visual feedback of the cursor trajectory were removed from the task for all groups, however, the 30° rotation of the cursor was still maintained. Participants were not informed of the continued rotation of the cursor and were instructed that their performance was still being monitored despite the lack of feedback (visual cursor trajectory and/or reinforcement). Additionally, the participants were informed “reach toward the target even without vision,” which is the same as a previous investigation that employed a similar methodology to the current study (Quattrocchi et al., 2018). After each trial during No Vision, the null feedback was presented to all groups at the same latency (Figure 1C). After each trial in all other conditions (Baseline, Washout, Readaptation), the same null feedback was also presented. The magnitude of feedback during the Adaptation condition was dependent on the amount of angular error that occurred in the trial performance and followed these criteria:

- ***Reward***: 4 points: hit the target; 3 points: <10° error; 2 points: <20° error; 1 point: <30° error; 0 points: ≥30° error.
- ***Punishment***: 0 points: hit the target; −1 point: <10° error; −2 points: <20° error; −3 points: <30° error; −4 points: ≥30° error.
- ***Null***: Points were replaced by two uninformative vertical lines.

All groups started with a total of zero points. Those in the Reward group earned positive points, while those in the Punishment group accrued negative points. Each point was equal to USD 0.02, a rule in which participants were not be made explicitly aware of. The Reward group began with USD 0.00 and earned money based on their performance during the Adaptation condition. The Punishment group began with USD 10.00 and lost money during the Adaptation condition. To control for payment and time of payment, participants in the Control group were randomly selected to receive USD 10.00 before the experiment and end the experiment with USD 6.00 or begin with USD 0.00 and end with USD 6.00. All participants were informed of the task goals by being read aloud a script before the start of the experiment. Additionally, participants in the Control group were given the instructions of either the Reward or Punishment groups, to control for the effects of the script.

### Visuomotor Rotation Task Analysis

Movement time was defined as the time from the first movement of the cursor outside of the starting circle to the termination of the movement in the direction of the target circle. Cartesian X and Y coordinates of the cursor were recorded and used to calculate our kinematic variables of interest. Absolute performance error was defined as the absolute maximum angular deviation of the movement of the cursor to the center of the target circle (Galea et al., 2011; Song et al., 2019). By using the absolute error we disregarded the direction of movement of the cursor (clockwise or counterclockwise) relative to the target and considered only the extent to which the cursor movement angle differs from the target angle (Christou et al., 2016). Performance errors exceeding 80° were excluded from the analysis which is similar to previous studies (Quattrocchi et al., 2018). Additionally, the Adaptation condition was divided into two learning stages: Early Learning was defined as the first 100 trials and Late Learning was defined as the last 100 trials. To best assess task retention, we compared performance error in Adaptation (Late Learning) to No Vision. Late Learning was considered when participants had learned the task and would be the best representation of the motor skill carried over into the No Vision (retention) condition.

### EEG Recording and Processing

Surface EEG data was recorded with a 28 channel Quik-Cap electrode system (Victoria, Australia) and NuAmps amplifier. Electrodes were placed according to the 10–20 system at sites FZ, FCZ, CZ, PZ, FP1, FP2, F3, F4, F7, F8, FT7, FT8, FC3, FC4, C3, C4, CP3, CP4, P3, P4, T3, T4, T5, T6, TP7, TP8, O1, O2, and ground placed on the participant’s right mastoid process. A saline solution was applied with a blunt tip syringe into the individual electrodes to lower electrical signal noise. The electrical impedance for each electrode was kept below 10 kΩ throughout the data collection. All recordings were sampled at 1,000 Hz, online band-pass filtered between 0.1–500 Hz, and notch filtered at 60 Hz.

All raw EEG data was exported and processed into MATLAB, using the EEGLAB toolbox (Delorme and Makeig, 2004). The raw data was downsampled from 1,000 Hz to 250 Hz and high-pass filtered at 1 Hz. Continuous data were segmented into time-locked data epochs. An initial visual inspection of the epochs was performed to remove trials containing artifacts. Then signal decomposition was performed using independent components analysis on each participant’s data utilizing the “runcia” procedure in EEGLAB. Additional trials containing artifacts were identified using the resultant components of the signal decomposition and were removed from the analysis. Components reflecting eye blinks and electromyography activity were removed by visual inspection. Participants that retained less than 75% of original trials were excluded from the analysis.

### Feedback-Related ERPs Computation and Analysis

Continuous EEG data were segmented into 2-s epochs (−500 ms to +1,500 ms), time-locked to the presentation of the feedback at 0 ms. Feedback-related ERPs were computed by averaging trials for each participant separately for the Adaptation (Early and Late Learning) and No Vision conditions. All ERPs were baseline corrected by subtracting the baseline activity 500 ms before feedback onset. Our analysis of feedback-related ERPs primarily focused on the channels FZ, FCZ, and PZ which is in line with previous research that investigated reinforcement feedback (Stürmer et al., 2011; Wischnewski et al., 2018; Palidis et al., 2019). The peak-to-peak amplitude of the feedback-related ERPs was calculated for each participant in Adaptation and No Vision conditions. Peak-to-peak amplitude was defined as the difference between the minimum peak 100 ms to 200 ms after the feedback onset and the maximal positive peak occurring between 250–600 ms after the feedback presentation (Palidis et al., 2019).

### Statistical Analysis

A Kruskal-Wallis H test was used to test for differences between groups on each BIS/BAS subcomponents (BAS FUN, BAS DRIVE, BAS REWARD RESPONSIVENESS, BIS).

Behavioral performance was analyzed by repeated-measures ANOVAs to assess for differences in movement time and performance error across different task conditions. Specifically, a 3 [Feedback Group] × 2 [Learning Stage (Early Learning, Late Learning)] and 3 [Feedback Group] × 2 [Task Condition (Late Learning, No Vision)] were utilized to test for differences in movement time and performance error averaged across all blocks in each condition. Also, a separate 3 [Feedback Group] × 2 [Task Condition (Adaptation Block 10, No Vision Block 1)] repeated-measures ANOVA was carried out to compare performance error during the end of the Adaptation condition (block 10) and the beginning of the No Vision condition (block 1), with each block contained 20 trials. Also, a one-way ANOVA was used to test for differences between groups in performance error during the beginning of the Adaptation condition.

EEG data were analyzed by repeated-measures ANOVAs to test for differences in peak-to-peak amplitude of feedback-related ERPs. Specifically, a 3 [Feedback Group] × 2 [Learning Stage (Early Learning, Late Learning)] and a 3 [Feedback Group] × 2 [Task Condition (Late Learning, No Vision)]. All frequentist statistical analysis was conducted with SPSS^®^ version 25 and set an *a priori* alpha level of 0.05. All non-significant ERP results from the frequentist statistics were followed-up by a Bayesian repeated-measures ANOVA with the same between and within-subjects factors and a default uniform prior of 0.5 for the fixed effects (r scale Cauchy prior width = 0.5; Wagenmakers et al., 2018). Bayes factors (*BF*_(10)_) were used to provide evidence for (*BF*_(10)_ of ≤ 0.33) or against the null (*BF*_(10)_ of ≥ 3.0) hypothesis. All Bayesian statistical analysis was conducted with JASP 0.11.1.0 (van Doorn et al., 2019).

Robust regression was utilized to examine the linear relationships between performance error and ERP measures (Palidis et al., 2019). This method assigns a lower weight to outlier data using an iteratively reweighted least-squares process. This analysis was implemented using the robust option of fitlm function in MATLAB.

## Results

### BAS/BIS Scale

A Kruskal-Wallis H test revealed no significant differences between groups on the BAS DRIVE (*H*_(2)_ = 4.029, *p* = 0.133), BAS FUN (*H*_(2)_ = 1.239, *p* = 0.538), BAS REWARD RESPONSIVENESS (*H*_(2)_ = 2.323, *p* = 0.313), and BIS (*H*_(2)_ = 1.426, *p* = 0.490) of the BAS/BIS scale (Table 1). Thus, all groups demonstrated similar sensitivity to reinforcement feedback.

**Table 1:** Average BAS/BIS scores (mean ± standard error) for each reinforcement group.

	BAS Drive	BAS FUN	BAS Reward	BIS
Reward	12.57 ± 0.41	12.35 ± 0.43	18.14 ± 0.34	21.57 ± 1.05
Punishment	11.50 ± 0.53	12.85 ± 0.43	18.14 ± 0.27	21.14 ± 0.86
Control	11.00 ± 0.49	12.14 ± 0.49	17.57 ± 0.27	20.35 ± 0.86

### Movement Time and Performance Error

**Figure 2:**
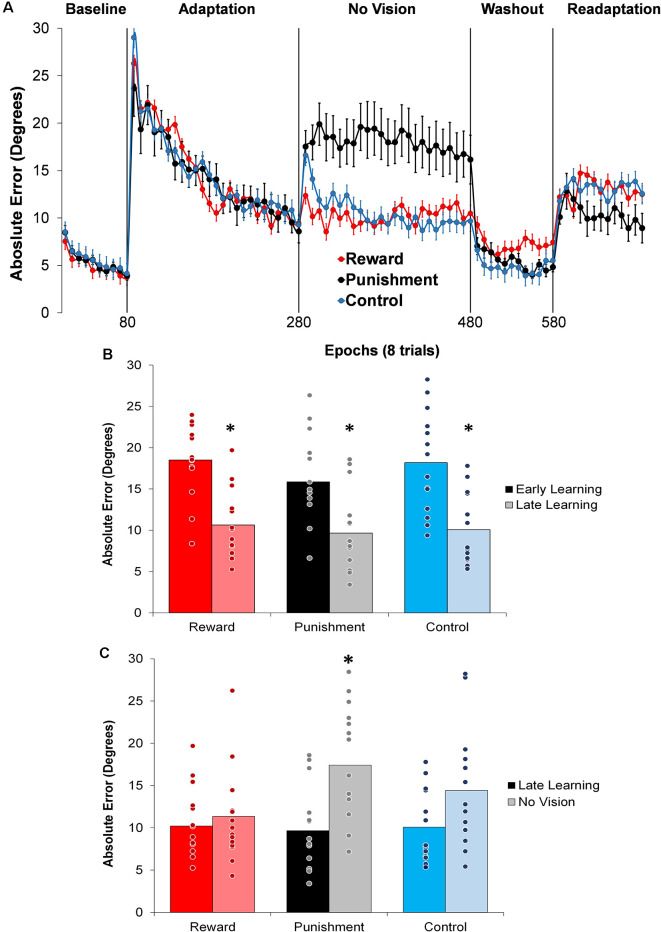
**(A)** Average absolute performance error across epochs of eight trials in each task condition for each of the groups. Represented as mean ± standard error. **(B)** Average absolute performance error for all groups during the Adaptation (Early and Late Learning) condition. *p < 0.05 compared to Early Learning. **(C)** Average absolute performance error for all groups during the Adaptation (Late Learning) and No Vision conditions. *p < 0.05 compared to Late Learning. Bars represent mean and dots represent the individual responses.

All groups performed and learned the task, which improved progressively with practice. Data on movement time are presented in Table 2 and data for performance error are depicted in Table 3 and Figure 2. No significant differences were detected for movement time in the Adaptation and No Vision conditions (*F*_(1,39)_ = 0.001, *p* = 0.992, $\eta_{\text{p}}^{2}$ = 0.001) and all groups demonstrated similar movement times (*F*_(2,39)_ = 0.303, *p* = 0.740, $\eta_{\text{p}}^{2}$ = 0.014). This finding indicates that all participants took similar times to move throughout each task condition regardless of the feedback group.

**Table 2:** Average movement time (mean ± standard error) for each group during each task condition.

	Movement Time (ms)					
	Baseline	Adaptation (Early Learning)	Adaptation (Late Learning)	No Vision	Washout	Readaptation
Reward	399.69 ± 6.93	386.35 ± 5.34	380.07 ± 2.31	380.79 ± 4.12	382.81 ± 4.46	377.55 ± 4.15
Punishment	397.89 ± 7.58	385.62 ± 5.38	376.93 ± 3.58	377.94 ± 5.59	375.88 ± 2.41	383.36 ± 6.48
Control	383.96 ± 5.49	379.59 ± 3.71	383.91 ± 6.49	382.22 ± 7.61	379.37 ± 4.34	375.18 ± 3.61

**Table 3:** Average performance error (mean ± standard error) for each group during each task condition.

	Performance Error (degrees)						
	Baseline	Adaptation (1st Block)	Adaptation (Early Learning)	Adaptation (Late Learning)	No Vision	Washout	Readaptation
Reward	5.22 ± 0.31	23.33 ± 1.21	18.55 ± 1.59*	11.54 ± 1.08	10.35 ± 1.87	7.14 ± 1.47	13.15 ± 1.68
Punishment	5.24 ± 0.44	18.32 ± 2.29^#^	15.85 ± 1.81*	10.57 ± 1.57	16.72 ± 2.36*^#^	5.29 ± 1.01	11.05 ± 1.67
Control	5.59 ± 0.41	24.47 ± 1.52	18.18 ± 2.09*	11.59 ± 1.41	10.69 ± 2.51	4.87 ± 1.09	13.38 ± 1.81

*p < 0.05 compared to Late Learning. ^#^p < 0.05 compared to Reward and Control groups.

During the Adaptation condition, all groups demonstrated a gradual reduction in performance error, indicating adequate movement recalibration to the 30° rotation. Upon a closer examination of the first block of Adaptation, our findings indicated that the Punishment group demonstrated lower error (*F*_(2,39)_ = 3.539, *p* = 0.039, $\eta_{\text{p}}^{2}$ = 0.154), thus faster learning, compared to Reward and Control, which is similar to previous studies (Song and Smiley-Oyen, 2017; Song et al., 2019; Figure 2A). Interestingly, the Punishment group also showed a higher standard deviation during this first block (*SD* = 8.58) [Reward (*SD* = 4.54) and Control (*SD* = 5.71)] which could be indicative of an increased motor exploration (Song et al., 2019). Our repeated measures ANOVA analysis revealed that after the first block all groups learned and maintain similar performance throughout the Adaptation condition. A significant main effect for Learning Stage (*F*_(1,39)_ = 81.431, *p* < 0.001, $\eta_{\text{p}}^{2}$ = 0.676) indicated that Late Learning had a lower performance error compared to Early Learning (Figure 2B). No significant differences were noted between groups (*F*_(1,39)_ = 0.647, *p* = 0.529, $\eta_{\text{p}}^{2}$ = 0.032).

The Punishment and Control groups demonstrated a decreased retention of the visuomotor rotation task indicated by a significant increase in performance error in the No Vision condition compared to the Adaptation condition (Late Learning). A significant Feedback Group × Task condition interaction (*F*_(2,39)_ = 3.549, *p* = 0.038, $\eta_{\text{p}}^{2}$ = 0.154) was noted between the end of the Adaptation (Block 10) and the beginning of the No Vision (Block 1). A test of simple effects revealed that both Punishment (*F*_(2,39)_ = 4.611, *p* > 0.001, $\eta_{\text{p}}^{2}$ = 0.453) and Control (*F*_(2,39)_ = 4.611, *p* = 0.038, $\eta_{\text{p}}^{2}$ = 0.191) had a significant decay in performance during the first block of the No Vision condition. However, Reward maintained their performance from Adaptation (Block 10) to No Vision (Block 1; *F*_(2,39)_ = 0.067, *p* = 0.798, $\eta_{\text{p}}^{2}$ = 0.002). Additionally, Punishment demonstrated significantly higher performance error compared to Reward (*F*_(2,39)_ = 7.289, *p* = 0.011, $\eta_{\text{p}}^{2}$ = 0.272) but not Control (*F*_(2,39)_ = 1.897, *p* = 0.0176, $\eta_{\text{p}}^{2}$ = 0.089) in the No Vision (Block 1). No significant differences were detected between Reward and Control groups (*F*_(2,39)_ = 1.749, *p* = 0.194, $\eta_{\text{p}}^{2}$ = 0.082).

The decreased in retention showed by the Punishment group was maintained during the No Vision condition. A significant Feedback Group × Task condition (Late Learning, No Vision) interaction (*F*_(2,39)_ = 3.349, *p* = 0.045, $\eta_{\text{p}}^{2}$ = 0.147) was noted for performance error when considering the average of the entire No Vision condition. A test of simple effects reveals that Punishment demonstrated a significant increase in performance error from Late Learning to No Vision (*F*_(2,39)_ = 7.624, *p* = 0.009, $\eta_{\text{p}}^{2}$ = 0.164). This decay was not seen in either in Reward (*F*_(2,39)_ = 0.285, *p* = 0.596, $\eta_{\text{p}}^{2}$ = 0.007) or Control (*F*_(2,39)_ = 0.071, *p* = 0.792, $\eta_{\text{p}}^{2}$ = 0.002) groups. Additionally, Punishment demonstrated a significant increase in performance error compared to Reward (*F*_(2,39)_ = 4.93, *p* = 0.032, $\eta_{\text{p}}^{2}$ = 0.151) and Control (*F*_(2,39)_ = 4.93, *p* = 0.042, $\eta_{\text{p}}^{2}$ = 0.119) groups during the No Vision condition, which suggest Punishment feedback interferes with the development of motor memory during the Adaptation condition (Figure 2C). Reward and Control groups performed similarly during the No Vision condition, indicating similar motor memory formation during task learning (*F*_(2,39)_ = 0.01, *p* = 0.906, $\eta_{\text{p}}^{2}$ = 0.001).

### Feedback-Related ERPs

Four participants did not meet the trial inclusion criteria, leaving the sample size of 38 for ERP analysis [Reward = 13 (seven female, six male), Punishment = 13 (seven female, six male), Control = 12 (six female, six male)].

**Figure 3:**
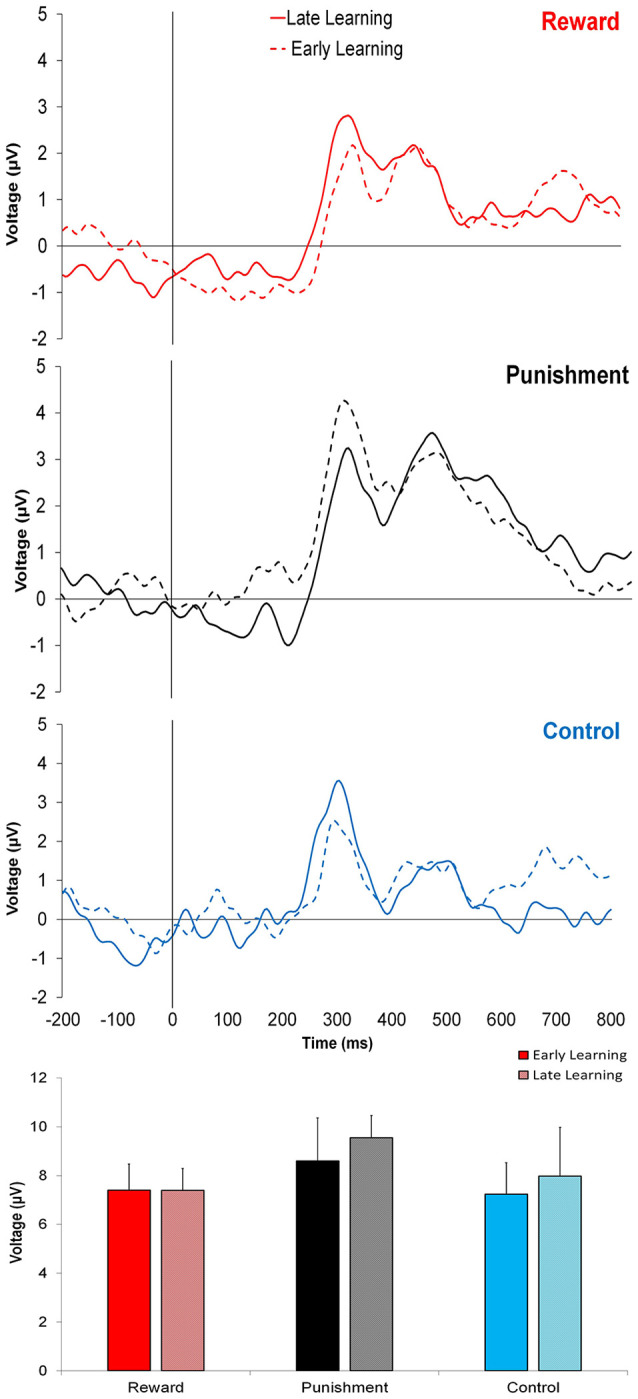
Grand average feedback-related event-related potentials (ERPs) for the three groups at the FCZ electrode during the Adaptation condition, Early Learning (dashed line), and Late Learning (solid line), with zero representing feedback presentation. The bottom bar graph represents the corresponding average ERP amplitude (mean ± standard error) for each group.

Figure 3 shows that learning the visuomotor rotation task did not impact the amplitude of feedback-related ERPs in any of the groups. No significant differences were detected for feedback-related ERP peak-to-peak amplitude between Early and Late Learning FCZ electrode with no significant differences between learning stages (*F*_(2,35)_ = 0.791, *p* = 0.380, $\eta_{\text{p}}^{2}$ = 0.022; Figure 3) or groups (*F*_(2,35)_ = 0.357, *p* = 0.702, $\eta_{\text{p}}^{2}$ = 0.021). Follow-up Bayesian analysis revealed similar results for learning stage (*BF*_(10)_ = 0.444) and groups (*BF*_(10)_ = 0.235).

Similar findings were noted for the FZ electrode with no significant differences between learning stages (*F*_(2,35)_ = 0.773, *p* = 0.385, $\eta_{\text{p}}^{2}$ = 0.022; *BF*_(10)_ = 0.371) or between groups (*F*_(2,35)_ = 1.816, *p* = 0.178, $\eta_{\text{p}}^{2}$ = 0.094; *BF*_(10)_ = 0.775).

This trend was also found in PZ electrode, with no significant differences between learning stages (*F*_(2,35)_ = 0.621, *p* = 0.436, $\eta_{\text{p}}^{2}$ = 0.017; *BF*_(10)_ = 0.642) or groups (*F*_(2,35)_ = 2.393, *p* = 0.106, $\eta_{\text{p}}^{2}$ = 0.121; *BF*_(10)_ = 0.304).

**Figure 4:**
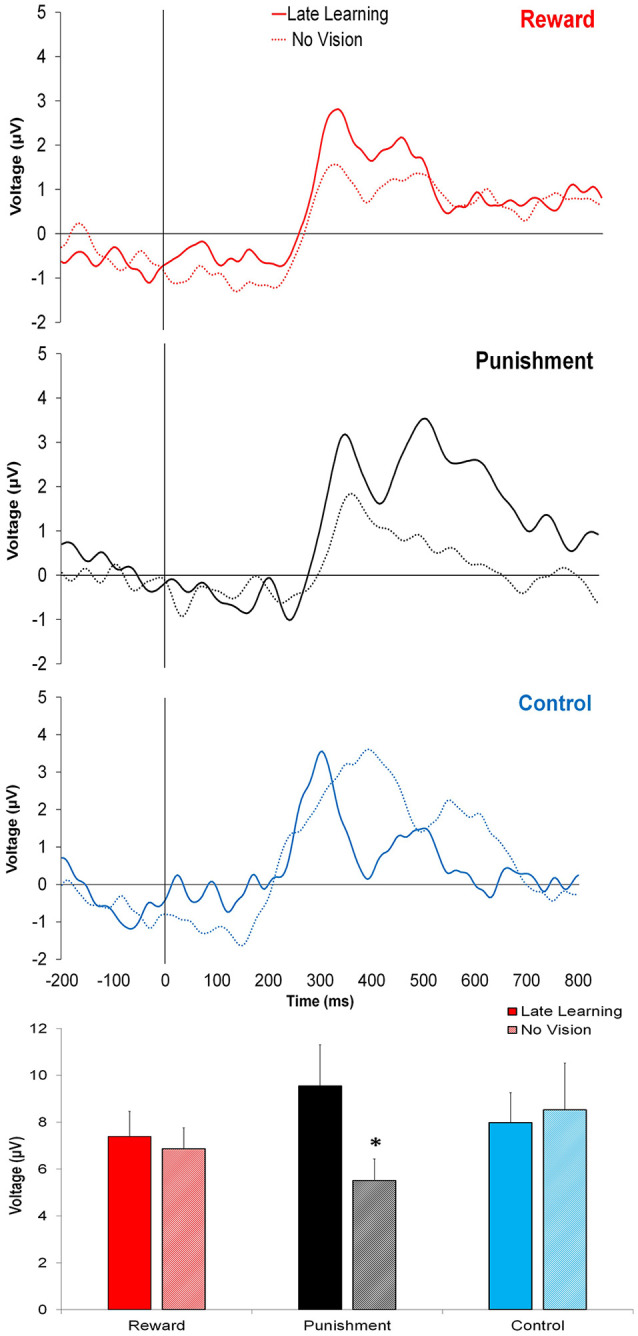
Grand average feedback-related ERPs for the three groups at the FCZ electrode during Adaptation (Late Learning; solid line) and No Vision (dotted line), with zero representing feedback presentation. The bottom bar graph represents the corresponding average ERP amplitude (mean ± standard error) for each group. *p < 0.05 compared to Late Learning.

**Figure 5:**
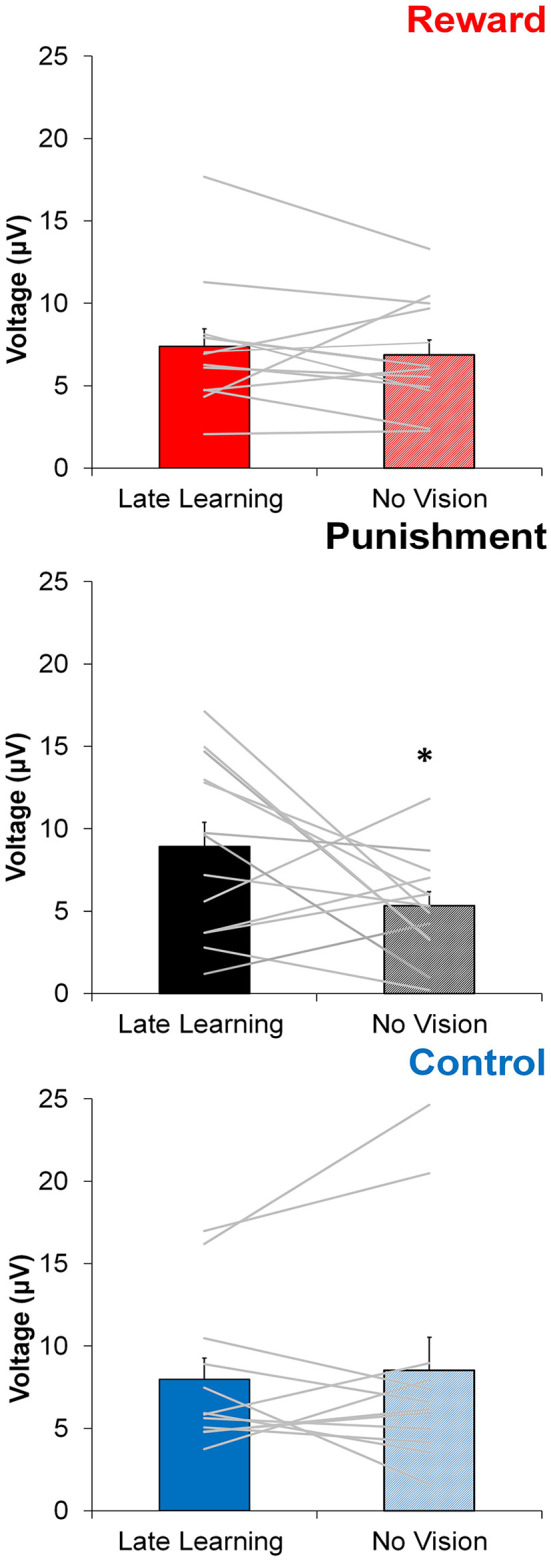
Average ERP amplitude (mean ± standard error) at the FCZ electrode during the Adaptation (Late Learning) and No Vision conditions for each group. Horizontal lines represent individual responses. *p < 0.05 compared to Late Learning.

In contrast to learning, the amplitude of feedback-related ERPs during Adaptation compared to No Vision was different among groups. A significant Group × Task Condition interaction (*F*_(2,35)_ = 3.361, *p* = 0.046, $\eta_{\text{p}}^{2}$ = 0.161) was found for feedback-related ERP peak-to-peak amplitude at the FCZ electrode. No significant differences were detected between groups within Adaptation (Late Learning; *F*_(2,35)_ = 1.078, *p* = 0.351, $\eta_{\text{p}}^{2}$ = 0.058) or No Vision condition (*F*_(2,35)_ = 1.468, *p* = 0.244, $\eta_{\text{p}}^{2}$ = 0.078; Figure 4). A test of simple effects revealed peak-to-peak amplitude for the Punishment group decreased from Adaptation (Late Learning) to No Vision (*F*_(2,35)_ = 7.687, *p* = 0.009, $\eta_{\text{p}}^{2}$ = 0.180; Figure 5). No significant differences were noted for Reward (*F*_(2,35)_ = 0.179, *p* = 0.675, $\eta_{\text{p}}^{2}$ = 0.005) or Control (*F*_(2,35)_ = 0.167, *p* = 0.686, $\eta_{\text{p}}^{2}$ = 0.005) groups.

**Figure 6:**
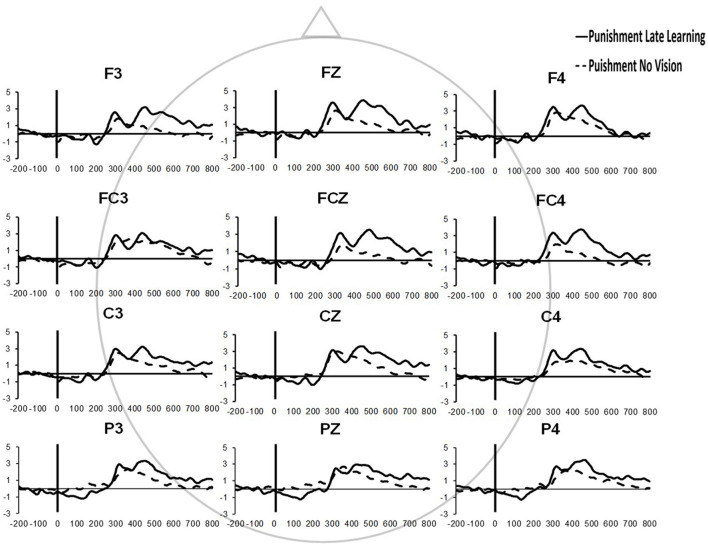
Grand average feedback-related ERPs of multiple channels for the punishment group during the Adaptation (Late Learning) and No Vision conditions.

Similar results were found for electrodes FZ and PZ. A significant Group × Task Condition interaction (*F*_(2,35)_ = 3.622, *p* = 0.037, $\eta_{\text{p}}^{2}$ = 0.171) was found for feedback-related ERP peak-to-peak amplitude at the FZ electrode. A test of simple effects revealed peak-to-peak amplitude for the Punishment group decreased from Adaptation (Late Learning) compared to No Vision (*F*_(2,35)_ = 11.921, *p* = 0.001, $\eta_{\text{p}}^{2}$ = 0.254). No differences were detected for Reward (*F*_(2,35)_ = 0.137, *p* = 0.714, $\eta_{\text{p}}^{2}$ = 0.004) and Control (*F*_(2,35)_ = 0.005, *p* = 0.944, $\eta_{\text{p}}^{2}$ > 0.001) groups. No significant differences were detected between groups within the Adaptation (Late Learning; *F*_(2,35)_ = 1.058, *p* = 0.358, $\eta_{\text{p}}^{2}$ = 0.057) or No Vision condition (*F*_(2,35)_ = 0.893, *p* = 0.419, $\eta_{\text{p}}^{2}$ = 0.049). A significant Group × Task Condition interaction (*F*_(2,35)_ = 3.566, *p* = 0.039, $\eta_{\text{p}}^{2}$ = 0.169) was found for feedback-related ERP peak-to-peak amplitude at the PZ electrode. A test of simple effects revealed peak-to-peak amplitude for the Punishment group decreased from Adaptation (Late Learning) to No Vision (*F*_(2,35)_ = 13.436, *p* = 0.001, $\eta_{\text{p}}^{2}$ = 0.277). No significant differences between groups were detected during Adaptation (Late Learning; *F*_(2,35)_ = 1.795, *p* = 0.181, $\eta_{\text{p}}^{2}$ = 0.093) or No Vision (*F*_(2,35)_ = 2.442, *p* = 0.102, $\eta_{\text{p}}^{2}$ = 0.122). Results for Fz and Pz electrodes in the punishment group are reflected in Figure 6.

Because we found that punishment feedback decreased the amplitude of ERPs and increased performance error during the No Vision condition, we tested whether ERPs amplitude during this condition predicted motor performance after punishment feedback by computing a Robust Regression analysis (see “Materials and Methods” section). The results of this analysis showed that there is no significant correlation between the No Vision peak-to-peak amplitude and No Vision performance error of the Punishment group for any of the electrodes measured (FCZ: *F*_(1,13)_ = 0.083, *p* = 0.777, *R*^2^ = 0.007; FZ: *F*_(1,13)_ = 0.078, *p* = 0.784, *R*^2^ = 0.011; PZ: *F*_(1,13)_ = 0.591, *p* = 0.458, *R*^2^ = 0.051).

## Discussion

The present study shows that punishment, but not reward, feedback modulates motor learning and feedback-related ERPs during the visuomotor rotation task. Subjects in the Punishment group showed a faster rate of adaptation upon first exposure to the 30° rotation and a stronger performance decay during the No Vision condition. These effects, not observed in the Reward group, suggest that punishment feedback improves motor learning and impairs motor retention. Critically, the impaired motor retention showed by the Punishment group was paralleled by a decrease in the amplitude of feedback-related ERPs during the No Vision condition, which further suggests that punishment feedback alters the brain processing involved in encoding motor memory.

### Punishment Feedback Improves Visuomotor Learning

Our study finds that punishment feedback enhances the rate of adaptation during motor learning which is in agreement with previous studies (Song and Smiley-Oyen, 2017; Song et al., 2019). This faster rate of adaptation could be associated with increased motor exploration during the task to optimize motor actions and therefore limit monetary loss (Song et al., 2019). This idea fits well in the frame of a “win-shift/lose-stay” decision making strategy (Worthy et al., 2013) for motor learning. According to this idea, the punishment would promote a more rapid change in reaching strategy to minimize loss, while reward and null feedbacks would not stimulate a strategy shift since the feedback for imperfect performance is non-aversive.

Interestingly, the effects of punishment enhancing the rate of adaptation shown in this study are limited to the initial exposure to the rotation, which is slightly different from previous similar studies (Galea et al., 2015; Song and Smiley-Oyen, 2017). These differences are not unexpected since our learning paradigm is not identical to the one utilized by these studies. Previous investigations have reported conflicting results regarding reinforcement feedback and the rate of motor adaptation. For instance, Galea et al. (2015) and Song and Smiley-Oyen (2017) demonstrated a sustained enhancement of adaptation in those that were punished. However, a more recent investigation found that punishment and reward affected adaptation similarly (Quattrocchi et al., 2018), while another study found that reward enhanced adaptation compared to punishment (Huang et al., 2018). Our results, taken together with previous research, suggest that the effects of reinforcement feedback on the rate of motor learning are complex and depend on the perturbation angle, the number of trials employed in the motor adaptation paradigm, and the procedural details regarding the context of the punishment (Song and Smiley-Oyen, 2017; Huang et al., 2018; Quattrocchi et al., 2018). Therefore, the effects of punishment feedback on motor learning are not settled and require further investigation.

As shown, all groups improved motor performance and decrease performance error during Late Learning compare to Early Learning. However, these changes in performance were not reflected in different feedback-related ERPs. Previous studies have presented decreases (Anguera et al., 2009; MacLean et al., 2015), increases (van der Helden et al., 2009) and no changes (Palidis et al., 2019) of feedback-related ERPs associated with different motor learning tasks indicating that the association between feedback-related ERPs and motor learning is still a matter of debate. Our study suggests that both Early and Late Learning require similar task engagement and brain processing during the visuomotor rotation task.

### Punishment Feedback Limits Visuomotor Retention and Changes Feedback-Related ERPs

Our results show that punishment feedback leads to worse retention of the motor task compared to reward and control feedback. These results are in line with studies showing that punishment feedback produces different effects on adaptation and retention of a motor task (Galea et al., 2015; Song and Smiley-Oyen, 2017; Quattrocchi et al., 2018) and supports the idea that the brain mechanisms contributing to motor learning and memory involve different neural pathways (Shadmehr and Krakauer, 2008; Huang et al., 2011; Galea et al., 2015; Haith et al., 2015; McDougle et al., 2015). Studies using transcranial magnetic stimulation (TMS) show that the activation (Galea et al., 2011; Spampinato et al., 2019) and inhibition (Hadipour-Niktarash et al., 2007) of the motor cortex (M1) enhances and decreases, respectively, motor memory without affecting motor adaptation, while the stimulation of the cerebellum enhances the rate of motor adaptation (Galea et al., 2011). In contrast to the effects of punishment, reward feedback did not change retention compared to null (Control) feedback, which is in agreement with recent evidence showing that null and reward feedbacks have similar retentions of a visuomotor task and that only the combination of both reward and M1 stimulation improves motor memory (Spampinato et al., 2019).

The mechanism underlying poor retention after punishment feedback may be indicative of the motor learning process employed by those in the Punishment group. It has been suggested that motor adaptation is comprised of “fast” and “slow” processes. “Fast” processes are characterized as being highly sensitive to error, rapidly changes behavior, and are subject to a quick decay in retention (Huang et al., 2018). Conversely, “slow” processes enhance retention and are more robust to decay but have slower adaptation (Smith et al., 2006; Ethier et al., 2008; Krakauer et al., 2019). As shown here, punishment feedback does fit this profile with faster learning during the Adaptation condition, and a stark decay in performance during the No Vision condition.

In contrast to punishment, we found large aftereffects (indicative of good motor retention) and lack of decay in the Reward group. These effects might be attributed to the usage of an explicit strategy, which is robust against changes in performance context (Codol et al., 2018). However, this possibility seems limited in our study given that participants were not informed to either maintain or remove any strategy. Also, we designed our visuomotor task with eight targets, which has been suggested to limit explicit strategy development (Galea et al., 2015). Alternatively, optimal motor retention could be driven by implicit mechanisms related to the visuomotor task. A recent study found large aftereffects in participants performing under No Vision conditions, unaware of the 30° counterclockwise rotation, and suggested that these aftereffects were driven by implicit processes with little contribution from explicit strategies (Modchalingam et al., 2019). Based on this evidence, our findings of large aftereffects in the Reward group, and to some extent in the Control group, could be due to a well-established implicit learning process facilitated by reward in combination with visual feedback. However, we will need further experiments to test this possibility.

We evaluated the amplitude of feedback-related ERPs to better understand how punishment and reward feedbacks are processed by the brain during the visuomotor rotation task. Specifically, we compared the amplitude of feedback-related ERPs during the Adaptation condition, when subjects received reinforcement feedback, and the No Vision condition, when no reinforcement or visual feedback was provided, and performance was memory-guided. Different from Control and Reward groups, subjects in the Punishment group showed a significantly lower feedback-related ERP amplitude during the No Vision condition compared to the Adaptation condition. This effect paralleled their lower performance (poor retention) during the No Vision condition and suggests that punishment feedback is impairing the neural processing involved in the formation of motor memory.

Studies in humans and animals suggest that changes in feedback-related ERPs reflect the stronger salience of punishment as motivational feedback (Walsh and Anderson, 2012; Jean-Richard-Dit-Bressel et al., 2018). Punishment feedback is, in part, processed by the anterior cingulate cortex (ACC) and feedback-related ERPs have been associated with the activity of ACC (Holroyd and Coles, 2002; De Martino et al., 2010; Walsh and Anderson, 2012). According to these studies, it can be expected that the activity of ACC will increase when punishment feedback is provided during the visuomotor adaptation task.

Importantly, ACC and M1 are functionally and anatomically connected (Paus, 2001; Wang et al., 2001; Williams et al., 2004; Wenderoth et al., 2005). Since M1 plays a key role in facilitating motor memory (Galea et al., 2011; Bostan and Strick, 2018), we hypothesize that an increased input from ACC to M1 during motor learning disrupts the neural encoding of motor memory and decreases motor retention as observed in the Punishment group. In support of this hypothesis are TMS studies showing that the disruption of M1 activity impairs motor memory (Hadipour-Niktarash et al., 2007). However, given the complex nature of ERP signals (Kappenman and Luck, 2011; Cohen, 2017), further studies are granted to determine how feedback-related ERPs during punishment are mechanistically associated with poor motor retention.

As an alternative, ACC activation by punishment feedback might also alter motor memory by inhibiting the activity of the cerebellum through ACC-cerebellum inputs and re-mapping the internal model. This alternative is supported by a neuroimaging study that found a negative correlation between ACC and cerebellar activation (Margulies et al., 2007). In contrast to this possibility, however, recent studies suggest that sensorimotor memory relies on explicit mechanisms independent of the cerebellum (McDougle et al., 2015; Holland et al., 2018; Codol et al., 2018), which is preferentially involved in calibrating the internal model for motor adaptation (Galea et al., 2011; Haith et al., 2015).

Several limitations can be noted for the current study. We used a smaller sample size compared to other behavioral studies (Galea et al., 2011; Codol et al., 2018) and absolute angular errors, instead of reach angle, to measure motor performance in the visuomotor task, which can limit the comparison of our results to previous studies. Additionally, we did not test if participants were utilizing an explicit strategy during the visuomotor task which has been shown to impact behavioral performance (Codol et al., 2018).

In conclusion, this study shows that punishment feedback enhances motor learning but impairs motor retention. Importantly, punishment-induced motor retention impairments were associated with changes in feedback-related ERPs which suggest that punishment feedback alters the neural processing involved in the formation of motor memory. These results expand previous studies by providing a neurophysiological correlate for the effects of punishment feedback and support the idea that motor learning and memory are, in part, independent processes that can be modulated differently by reinforcement feedback. Based on ours and previous studies, we propose that punishment is salient motivational feedback that impairs motor memory by interfering with M1 activity through, presumably, ACC activation. Our results are relevant in the context of rehabilitation and training by discouraging the use of punishment feedback to improve motor learning in the long-term.

## Data Availability Statement

The datasets generated for this study are available on request to the corresponding author.

## Ethics Statement

The studies involving human participants were reviewed and approved by University of Mississippi Institutional Review Board. Written informed consent to participate in this study was provided by the participants’ legal guardian/next of kin.

## Author Contributions

CH designed the experiments, collected the data, analyzed the data, and wrote the manuscript. MS designed the experiments and analyzed the data. DW designed the experiments and collected the data. AD designed the experiments, analyzed the data, and wrote the manuscript.

## Conflict of Interest

The authors declare that the research was conducted in the absence of any commercial or financial relationships that could be construed as a potential conflict of interest.

## References

[B1] Abe M. Schambra H. Wassermann E. M. Luckenbaugh D. Schweighofer N. Cohen L. G. (2011). Reward improves long-term retention of a motor memory through induction of offline memory gains. Curr. Biol. 21, 557–562. 10.1016/j.cub.2011.02.030 21419628 PMC3075334

[B2] Anguera J. A. Seidler R. D. Gehring W. J. (2009). Changes in performance monitoring during sensorimotor adaptation. J. Neurophysiol. 102, 1868–1879. 10.1152/jn.00063.2009 19605614 PMC2746769

[B3] Batcho C. S. Gagné M. Bouyer L. J. Roy J. S. Mercier C. (2016). Impact of online visual feedback on motor acquisition and retention when learning to reach in a force field. Neuroscience 337, 267–275. 10.1016/j.neuroscience.2016.09.020 27646292

[B4] Bostan A. C. Strick P. L. (2018). The basal ganglia and the cerebellum: nodes in an integrated network. Nat. Rev. Neurosci. 19, 338–350. 10.1038/s41583-018-0002-7 29643480 PMC6503669

[B104] Carver C. S. White T. L. (1994). Behavioral inhibition, behavioral activation, and affective responses to impending reward and punishment: the BIS/BAS scales. J. Personal. Soc. Psychol. 67, 319–333. 10.1037/0022-3514.67.2.319

[B105] Christou A. I. Miall R. C. McNab F. Galea F. (2016). Individual differences in explicit and implicit visuomotor learning and working memory capacity. Nat. Scienti. Reports 6:36633 10.1038/srep36633 PMC5100548 27824129

[B5] Codol O. Holland P. J. Galea J. M. (2018). The relationship between reinforcement and explicit control during visuomotor adaptation. Sci. Rep. 8:9121. 10.1038/s41598-018-27378-1 29904096 PMC6002524

[B6] Cohen M. X. (2017). Where does EEG come from and what does it mean? Trends Neurosci. 40, 208–218. 10.1016/j.tins.2017.02.004 28314445

[B7] Cohen M. X. Ranganath C. (2007). Reinforcement learning signals predict future decisions. J. Neurosci. 27, 371–378. 10.1523/jneurosci.4421-06.2007 17215398 PMC6672075

[B106] Delorme A. Makeig S. (2004). EEGLAB: an open source toolbox for analysis of single-trial EEG dynamics including independent component analysis. J. Neurosci. Met. 134, 9–21. 10.1016/j.jneumeth.2003.10.009 15102499

[B8] De Martino B. Camerer C. F. Adolphs R. (2010). Amygdala damage eliminates monetary loss aversion. Proc. Natl. Acad. Sci. U S A 107, 3788–3792. 10.1073/pnas.0910230107 20142490 PMC2840433

[B9] Ethier V. Zee D. S. Shadmehr R. (2008). Spontaneous recovery of motor memory during saccade adaptation. J. Neurophysiol. 99, 2577–2583. 10.1152/jn.00015.2008 18353917 PMC2733835

[B10] Ferdinand N. K. Opitz B. (2014). Different aspects of performance feedback engage different brain areas: Disentangling valence and expectancy in feedback processing. Sci. Rep. 4:5986. 10.1038/srep05986 25100234 PMC5380015

[B11] Galea J. M. Mallia E. Rothwell J. Diedrichsen J. (2015). The dissociable effects of punishment and reward on motor learning. Nat. Neurosci. 18, 597–602. 10.1038/nn.3956 25706473

[B103] Galea J. M. Vazquez A. Pasricha N. Orban de Xivry J. J. Celnik P. (2011). Dissociating the roles of the cerebellum and motor cortex during adaptive learning: the motor cortex retains what the cerebellum learns. Cerebral Cortex 21, 1761–1770. 10.1093/cercor/bhq246 21139077 PMC3138512

[B12] Gehring W. J. Willoughby A. R. (2002). The medial frontal cortex and the rapid processing of monetary gains and losses. Science 295, 2279–2282. 10.1126/science.1066893 11910116

[B13] Hadipour-Niktarash A. Lee C. K. Desmond J. E. Shadmehr R. (2007). Impairment of retention but not acquisition of a visuomotor skill through time-dependent disruption of primary motor cortex. J. Neurosci. 27, 13413–13419. 10.1523/jneurosci.2570-07.2007 18057199 PMC6673085

[B14] Haith A. M. Huberdeau D. M. Krakauer J. W. (2015). The influence of movement preparation time on the expression of visuomotor learning and savings. J. Neurosci. 35, 5109–5117. 10.1523/jneurosci.3869-14.2015 25834038 PMC6705405

[B15] Hajack G. Moser J. Holroyd C. B. Simons R. F. (2006). The feedback-related negativity reflects the binary evaluation of good versus bad outcomes. Biol. Psychol. 71, 148–154. 10.1016/j.biopsycho.2005.04.001 16005561

[B16] Hester R. Murphy K. Brown F. L. Skilleter A. J. (2010). Punishing an error improves learning: the influence of punishment magnitude on error-related neural activity and subsequent learning. J. Neurosci. 30, 15600–15607. 10.1523/jneurosci.2565-10.2010 21084615 PMC6633683

[B102] Hinder M. R. Riek S. Tresilian J. R. de Rugy A. Carson R. G. (2010). Realtime error detection but not error correction drives automatic visuomotor adaptation. Experiment. Brian Res. 201, 197–207. 10.1007/s00221-009-2025-9 19830412

[B17] Holland P. Codol O. Galea J. M. (2018). Contribution of explicit processes to reinforcement-based motor learning. J. Neurophysiol. 119, 2241–2255. 10.1152/jn.00901.2017 29537918 PMC6032115

[B18] Holroyd C. B. Coles M. G. (2002). The neural basis of human error processing: reinforcement learning, dopamine, and the error-related negativity. Psychol. Rev. 109, 679–709. 10.1037/0033-295X.109.4.679 12374324

[B19] Holroyd C. B. Larsen J. T. Cohen J. D. (2004). Context dependence of the event-related brain potential associated with reward and punishment. Psychophysiology 41, 245–253. 10.1111/j.1469-8986.2004.00152.x 15032989

[B21] Huang V. S. Haith A. Mazzoni P. Krakauer J. W. (2011). Rethinking motor learning and savings in adaptation paradigms: model-free memory for successful actions combines with internal models. Neuron 70, 787–801. 10.1016/j.neuron.2011.04.012 21609832 PMC3134523

[B20] Huang J. Hegele M. Billino J. (2018). Motivational modulation of age-related effects on reaching adaptation. Front. Psychol. 9:2285. 10.3389/fpsyg.2018.02285 30515126 PMC6255948

[B22] Huang Y. Yu R. (2014). The feedback-related negativity reflects “more or less” prediction error in appetitive and aversive conditions. Front. Neurosci. 8:108. 10.3389/fnins.2014.00108 24904254 PMC4033096

[B23] Izawa J. Shadmehr R. (2011). Learning from sensory and reward prediction errors during motor adaptation. PLoS Comput. Biol. 7:e1002012. 10.1371/journal.pcbi.1002012 21423711 PMC3053313

[B24] Jean-Richard-Dit-Bressel P. Killcross S. McNally G. P. (2018). Behavioral and neurobiological mechanisms of punishment: implications for psychiatric disorders. Neuropsychopharmacology 43, 1639–1650. 10.1038/s41386-018-0047-3 29703994 PMC6006171

[B25] Kappenman E. S. Luck S. J. (Eds). (2011). “ERP components: the ups and downs of brainwave recordings,” in The Oxford Handbook of Event-Related Potential Components, (New York, NY: Oxford University Press), 3–30. 10.31234/osf.io/yqxfr

[B26] Krakauer J. W. Hadjiosif A. M. Xu J. Wong A. L. Haith A. M. (2019). Motor learning. Compr. Physiol. 9, 613–663. 10.1002/cphy.c170043 30873583

[B27] Leow L. A. De Rugy A. Marinovic W. Riek S. Carroll T. J. (2016). Savings for visuomotor adaptation require prior history of error, not prior repetition of successful actions. J. Neurophysiol. 116, 1603–1614. 10.1152/jn.01055.2015 27486109 PMC5144718

[B28] MacLean S. J. Hassall C. D. Ishigami Y. Krigolson O. E. Eskes G. A. (2015). Using brain potentials to understand prism adaptation: the error-related negativity and the P300. Front. Hum. Neurosci. 9:335. 10.3389/fnhum.2015.00335 26124715 PMC4464183

[B29] Margulies D. S. Kelly A. C. Uddin L. Q. Biswal B. B. Castellanos F. X. Milham M. P. (2007). Mapping the functional connectivity of anterior cingulate cortex. NeuroImage 37, 579–588. 10.1016/j.neuroimage.2007.05.019 17604651

[B30] Marko M. K. Haith A. M. Harran M. D. Shadmehr R. (2012). Sensitivity to prediction error in reach adaptation. J. Neurophysiol. 108, 1752–1763. 10.1152/jn.00177.2012 22773782 PMC3774589

[B31] Mazzoni P. Krakauer J. W. (2006). An implicit plan overrides an explicit strategy during visuomotor adaptation. J. Neurosci. 26, 3642–3645. 10.1523/jneurosci.5317-05.2006 16597717 PMC6674132

[B32] McDougle S. D. Bond K. M. Taylor J. A. (2015). Explicit and implicit processes constitute the fast and slow processes of sensorimotor learning. J. Neurosci. 35, 9568–9579. 10.1523/jneurosci.5061-14.2015 26134640 PMC4571499

[B33] Modchalingam S. Vachon C. M. Marius‘t Hart B. Henriques D. Y. (2019). The effects of awareness of the perturbation during motor adaptation on hand localization. PLoS One 14:e0220884. 10.1371/journal.pone.0220884 31398227 PMC6688819

[B34] Nieuwenhuis S. Holroyd C. B. Mol N. Coles M. G. (2004). Reinforcement-related brain potentials from medial frontal cortex: origins and functional significance. Neurosci. Biobehav. Rev. 28, 441–448. 10.1016/j.neubiorev.2004.05.003 15289008

[B35] Nikooyan A. A. Ahmed A. A. (2014). Reward feedback accelerates motor learning. J. Neurophysiol. 113, 633–646. 10.1152/jn.00032.2014 25355957

[B36] Oldfield R. C. (1971). The assessment and analysis of handedness: the Edinburgh inventory. Neuropsychologia 9, 97–113. 10.1016/0028-3932(71)90067-4 5146491

[B37] Palidis D. J. Cashaback J. G. Gribble P. L. (2019). Neural signatures of reward and sensory error feedback processing in motor learning. J. Neurophysiol. 121, 1561–1574. 10.1152/jn.00792.2018 30811259 PMC6485737

[B38] Paus T. (2001). Primate anterior cingulate cortex: where motor control, drive and cognition interface. Nat. Rev. Neurosci. 2, 417–424. 10.1038/35077500 11389475

[B39] Quattrocchi G. Monaco J. Ho A. Irmen F. Strube W. Ruge D.. (2018). Pharmacological dopamine manipulation does not alter reward-based improvements in memory retention during a visuomotor adaptation task. eNeuro 5:ENEURO.0453-17.2018. 10.1523/eneuro.0453-17.2018 30027109 PMC6051592

[B40] San Martín R. (2012). Event-related potential studies of outcome processing and feedback-guided learning. Front. Hum. Neurosci. 6:304. 10.3389/fnhum.2012.00304 23162451 PMC3491353

[B41] Schuermann B. Endrass T. Kathmann N. (2012). Neural correlates of feedback processing in decision-making under risk. Front. Hum. Neurosci. 6:204. 10.3389/fnhum.2012.00204 22783182 PMC3390593

[B42] Schween R. Hegele M. (2017). Feedback delay attenuates implicit but facilitates explicit adjustments to a visuomotor rotation. Neurobiol. Learn. Mem. 140, 124–133. 10.1016/j.nlm.2017.02.015 28257877

[B101] Schween R. Taube W. Gollhofer A. Leukel C. (2014). Online and post-trial feedback differentially affect implicit adaptation to a visuomtor rotation. Experiment. Brian Res. 232, 3007–3013. 10.1007/s00221-014-3992-z 24854018

[B43] Shabbott B. A. Sainburg R. L. (2010). Learning a visuomotor rotation: simultaneous visual and proprioceptive information is crucial for visuomotor remapping. Exp. Brain Res. 203, 75–87. 10.1007/s00221-010-2209-3 20237773 PMC3702748

[B45] Shadmehr R. De Xivry J. J. O. Xu-Wilson M. Shih T. Y. (2010). Temporal discounting of reward and the cost of time in motor control. J. Neurosci. 30, 10507–10516. 10.1523/jneurosci.1343-10.2010 20685993 PMC2926660

[B44] Shadmehr R. Krakauer J. W. (2008). A computational neuroanatomy for motor control. Exp. Brain Res. 185, 359–381. 10.1007/s00221-008-1280-5 18251019 PMC2553854

[B107] Smith M. A. Ghazizadeh A. Shadmehr R. (2006). Interacting adaptive processes with different timescales underlie short-term motor learning. PLoS Biol. 4:e179 10.1371/journal.pbio.0040179 16700627 PMC1463025

[B47] Song Y. Lu S. Smiley-Oyen A. L. (2019). Differential motor learning *via* reward and punishment. Q. J. Exp. Psychol. 73, 249–259. 10.1177/1747021819871173 31382855

[B46] Song Y. Smiley-Oyen A. L. (2017). Probability differently modulating the effects of reward and punishment on visuomotor adaptation. Exp. Brain Res. 235, 3605–3618. 10.1007/s00221-017-5082-5 28887626

[B48] Spampinato D. Satar Z. Rothwell J. (2019). Combining reward and M1 transcranial direct current stimulation enhances the retention of newly learnt sensorimotor mappings. Brain Stimul. 12, 1205–1212. 10.1016/j.brs.2019.05.015 31133478 PMC6709642

[B49] Stürmer B. Nigbur R. Schacht A. Sommer W. (2011). Reward and punishment effects on error processing and conflict control. Front. Psychol. 2:335. 10.3389/fpsyg.2011.00335 22110464 PMC3217218

[B50] Taylor J. A. Ivry R. B. (2014). Cerebellar and prefrontal cortex contributions to adaptation, strategies and reinforcement learning. Prog. Brain Res. 2010, 217–253. 10.1016/b978-0-444-63356-9.00009-1 24916295 PMC4118688

[B51] Therrien A. S. Wolpert D. M. Bastian A. J. (2016). Effective reinforcement learning following cerebellar damage requires a balance between exploration and motor noise. Brain 139, 101–114. 10.1093/brain/awv329 26626368 PMC4949390

[B52] Torrecillos F. Albouy P. Brochier T. Malfait N. (2014). Does the processing of sensory and reward-prediction errors involve common neural resources? Evidence from a frontocentral negative potential modulated by movement execution errors. J. Neurosci. 34, 4845–4856. 10.1523/jneurosci.4390-13.2014 24695704 PMC6802716

[B53] Ullsperger M. Danielmeier C. Jocham G. (2014). Neurophysiology of performance monitoring and adaptive behavior. Physiol. Rev. 94, 35–79. 10.1152/physrev.00041.2012 24382883

[B54] van der Helden J. Boksem M. A. Blom J. H. (2009). The importance of failure: feedback-related negativity predicts motor learning efficiency. Cereb. Cortex 20, 1596–1603. 10.1016/s1053-8119(09)71967-6 19840974

[B55] van Doorn J. van den Bergh D. Bohm U. Dablander F. Derks K. Draws T. (2019). The JASP guidelines for conducting and reporting a Bayesian analysis. PsyArXiv 1, 1–38. 10.31234/osf.io/yqxfr PMC8219590 33037582

[B56] Wächter T. Lungu O. V. Liu T. Willingham D. T. Ashe J. (2009). Differential effect of reward and punishment on procedural learning. J. Neurosci. 29, 436–443. 10.1523/jneurosci.4132-08.2009 19144843 PMC2765863

[B57] Wagenmakers E. J. Love J. Marsman M. Jamil T. Ly A. Verhagen J.. (2018). Bayesian inference for psychology. Part II: Example applications with JASP. Psychon. Bull. Rev. 25, 58–76. 10.3758/s13423-017-1323-7 28685272 PMC5862926

[B58] Walsh M. M. Anderson J. R. (2012). Learning from experience: event-related potential correlates of reward processing, neural adaptation and behavioral choice. Neurosci. Biobehav. Rev. 36, 1870–1884. 10.1016/j.neubiorev.2012.05.008 22683741 PMC3432149

[B59] Wang Y. Shima K. Sawamura H. Tanji J. (2001). Spatial distribution of cingulate cells projecting to the primary, supplementary, and pre-supplementary motor areas: a retrograde multiple labeling study in the macaque monkey. Neurosci. Res. 39, 39–49. 10.1016/s0168-0102(00)00198-x 11164252

[B60] Wenderoth N. Debaere F. Sunaert S. Swinnen S. P. (2005). The role of anterior cingulate cortex and precuneus in the coordination of motor behavior. Eur. J. Neurosci. 22, 235–246. 10.1111/j.1460-9568.2005.04176.x 16029213

[B61] Williams Z. M. Bush G. Rauch S. L. Cosgrove G. R. Eskandar E. N. (2004). Human anterior cingulate neurons and the integration of monetary reward with motor responses. Nat. Neurosci. 7, 1370–1375. 10.1038/nn1354 15558064

[B62] Wischnewski M. Bekkering H. Schutter D. J. (2018). Frontal cortex electrophysiology in reward-and punishment-related feedback processing during advice-guided decision making: an interleaved EEG-DC stimulation study. Cogn. Affect. Behav. Neurosci. 18, 249–262. 10.3758/s13415-018-0566-8 29380293 PMC5889418

[B63] Worthy D. A. Hawthorne M. J. Otto A. R. (2013). Heterogeneity of strategy use in the Iowa gambling task: a comparison of win-stay/lose-shift and reinforcement learning models. Psychon. Bull. Rev. 20, 364–371. 10.3758/s13423-012-0324-9 23065763

[B64] Wrase J. Kahnt T. Schlagenhauf F. Beck A. Cohen M. X. Knutson B.. (2007). Different neural systems adjust motor behavior in response to reward and punishment. NeuroImage 36, 1253–1262. 10.1016/j.neuroimage.2007.04.001 17521924

[B580] Yeung N. Sanfey A. G. (2004). Independent coding of reward magnitude and valence in the human brain. J. Neurosci. 24, 6258–6264. 10.1523/JNEUROSCI.4537-03.2004 15254080 PMC6729539

